# Supplementary material for: Heparin-based hydrogel scaffolding alters the transcriptomic profile and increases the chemoresistance of MDA-MB-231 triple-negative breast cancer cells
Source: Biomater Sci. 2020 Feb 13;8(10):2786–96. doi: 10.1039/c9bm01481k (PMC7497406; doi:10.1039/c9bm01481k)
Supplement: Supplementary file 2 [file BM-008-C9BM01481K-s002.zip › Supplementary File 4/HP-B hydrogel + EGF (s)vControl/Pathways/my_analysis.Gsea.1545201170987/HALLMARK_REACTIVE_OXIGEN_SPECIES_PATHWAY.html]

Details for gene set HALLMARK\_REACTIVE\_OXIGEN\_SPECIES\_PATHWAY[GSEA]

|  || Dataset | expr.class.cls#HYDEGF\_versus\_CONTROL.class.cls#HYDEGF\_versus\_CONTROL\_repos |
| Phenotype | class.cls#HYDEGF\_versus\_CONTROL\_repos |
| Upregulated in class | CONTROL |
| GeneSet | HALLMARK\_REACTIVE\_OXIGEN\_SPECIES\_PATHWAY |
| Enrichment Score (ES) | -0.502627 |
| Normalized Enrichment Score (NES) | -1.8921367 |
| Nominal p-value | 0.0 |
| FDR q-value | 5.957747E-4 |
| FWER p-Value | 0.005 |
Table: GSEA Results Summary

  

Fig 1: Enrichment plot: HALLMARK\_REACTIVE\_OXIGEN\_SPECIES\_PATHWAY      
 Profile of the Running ES Score & Positions of GeneSet Members on the Rank Ordered List

  

| PROBE | DESCRIPTION (from dataset) | GENE SYMBOL | GENE\_TITLE | RANK IN GENE LIST | RANK METRIC SCORE | RUNNING ES | CORE ENRICHMENT || 1 | HHEX | na |  |  | 444 | 2.181 | 0.0262 | No |
| 2 | ERCC2 | na |  |  | 455 | 2.172 | 0.0748 | No |
| 3 | TXNRD2 | na |  |  | 1568 | 1.403 | 0.0485 | No |
| 4 | SCAF4 | na |  |  | 2867 | 1.036 | 0.0042 | No |
| 5 | ABCC1 | na |  |  | 3065 | 0.988 | 0.0163 | No |
| 6 | STK25 | na |  |  | 3666 | 0.872 | 0.0047 | No |
| 7 | HMOX2 | na |  |  | 4704 | 0.706 | -0.0334 | No |
| 8 | LSP1 | na |  |  | 5501 | 0.576 | -0.0619 | No |
| 9 | SBNO2 | na |  |  | 6736 | 0.405 | -0.1172 | No |
| 10 | NQO1 | na |  |  | 7079 | 0.362 | -0.1268 | No |
| 11 | CDKN2D | na |  |  | 7312 | 0.328 | -0.1315 | No |
| 12 | OXSR1 | na |  |  | 7659 | 0.281 | -0.1432 | No |
| 13 | GCLC | na |  |  | 8925 | 0.131 | -0.2063 | No |
| 14 | EGLN2 | na |  |  | 9156 | 0.108 | -0.2159 | No |
| 15 | FES | na |  |  | 9236 | 0.098 | -0.2178 | No |
| 16 | G6PD | na |  |  | 10053 | 0.012 | -0.2601 | No |
| 17 | PFKP | na |  |  | 10539 | -0.041 | -0.2845 | No |
| 18 | GCLM | na |  |  | 10721 | -0.067 | -0.2924 | No |
| 19 | TXNRD1 | na |  |  | 11750 | -0.196 | -0.3416 | No |
| 20 | MBP | na |  |  | 11814 | -0.206 | -0.3403 | No |
| 21 | SRXN1 | na |  |  | 12662 | -0.307 | -0.3775 | No |
| 22 | FTL | na |  |  | 13490 | -0.412 | -0.4114 | No |
| 23 | GLRX2 | na |  |  | 13865 | -0.469 | -0.4203 | No |
| 24 | MSRA | na |  |  | 14310 | -0.522 | -0.4317 | No |
| 25 | GPX4 | na |  |  | 14401 | -0.538 | -0.4242 | No |
| 26 | PRDX4 | na |  |  | 15690 | -0.760 | -0.4742 | No |
| 27 | JUNB | na |  |  | 15969 | -0.814 | -0.4703 | No |
| 28 | GSR | na |  |  | 16589 | -0.967 | -0.4807 | Yes |
| 29 | GPX3 | na |  |  | 16764 | -1.015 | -0.4669 | Yes |
| 30 | NDUFS2 | na |  |  | 16865 | -1.045 | -0.4484 | Yes |
| 31 | TXN | na |  |  | 17008 | -1.092 | -0.4311 | Yes |
| 32 | PRNP | na |  |  | 17039 | -1.101 | -0.4078 | Yes |
| 33 | PDLIM1 | na |  |  | 17405 | -1.216 | -0.3993 | Yes |
| 34 | PRDX2 | na |  |  | 18021 | -1.496 | -0.3975 | Yes |
| 35 | PRDX1 | na |  |  | 18026 | -1.499 | -0.3638 | Yes |
| 36 | PRDX6 | na |  |  | 18067 | -1.524 | -0.3314 | Yes |
| 37 | ATOX1 | na |  |  | 18310 | -1.694 | -0.3057 | Yes |
| 38 | NDUFA6 | na |  |  | 18511 | -1.845 | -0.2744 | Yes |
| 39 | NDUFB4 | na |  |  | 18632 | -1.965 | -0.2362 | Yes |
| 40 | GLRX | na |  |  | 18639 | -1.972 | -0.1918 | Yes |
| 41 | CAT | na |  |  | 18668 | -2.014 | -0.1477 | Yes |
| 42 | SOD1 | na |  |  | 18910 | -2.370 | -0.1066 | Yes |
| 43 | SOD2 | na |  |  | 18995 | -2.569 | -0.0529 | Yes |
| 44 | MGST1 | na |  |  | 19072 | -2.801 | 0.0066 | Yes |
Table: GSEA details [plain text format]

  

Fig 2: HALLMARK\_REACTIVE\_OXIGEN\_SPECIES\_PATHWAY      
 Blue-Pink O' Gram in the Space of the Analyzed GeneSet

  

Fig 3: HALLMARK\_REACTIVE\_OXIGEN\_SPECIES\_PATHWAY: Random ES distribution      
 Gene set null distribution of ES for **HALLMARK\_REACTIVE\_OXIGEN\_SPECIES\_PATHWAY**
